# Distribution of Four Vole Species through the Barn Owl *Tyto alba* Diet Spectrum: Pattern Responses to Environmental Gradients in Intensive Agroecosystems of Central Greece

**DOI:** 10.3390/life13010105

**Published:** 2022-12-30

**Authors:** Vasileios Bontzorlos, Konstantinos Vlachopoulos, Anastasios Xenos

**Affiliations:** 1 TYTO—Association for the Management and Conservation of Biodiversity in Agricultural Ecosystems, 41335 Larisa, Greece; 2 Department of Agriculture, Crop Production and Rural Environment, University of Thessaly, 38446 Volos, Greece

**Keywords:** voles, *Microtus*, Greece, Thessaly, Barn Owl, multivariate analysis

## Abstract

**Simple Summary:**

Voles of the genus *Microtus* represent one of the most speciose mammalian genera in the Holarctic. Moreover, they are also widespread in the European agricultural landscape. In addition, they are the most common vertebrate pests in European agriculture, causing extensive annual damages to a large variety of crops and exposing humans to an increased risk of pathogen transmission. In order to propose mitigation and possible management measures, it is necessary to record the initial abundance and distribution patterns of vole species within complex ecosystems. Considering the large extensions occupied by agroecosystems and the demanding but small-scale application of live trapping, an optimal method to initiate small mammal monitoring on large spatial scales is through Barn Owl *Tyto alba* diet analysis. Barn Owls prey almost exclusively on small mammals and predominantly on voles; thus, they can be used as an ideal proxy for the distribution and abundance patterns of small mammals in the field. In our study, we studied the diet of Barn Owls over a total range of 3000 sq. km for two continuous years through pellet analysis (more than 10,000 pellets) and identified the distribution and abundance patterns of four vole species with respect to different environmental gradients.

**Abstract:**

Voles are the most common vertebrate pests in European agriculture. Identifying their distribution and abundance patterns provides valuable information for future management. Barn Owl diet analysis is one of the optimum methods used to record small mammal distribution patterns on large spatial scales. From 2003 to 2005, a total of 10,065 Barn Owl pellets were collected and analyzed from 31 breeding sites in the largest agroecosystem in Greece, the Thessaly plains. A total of 29,061 prey items were identified, offering deep insight into small mammal distribution, specifically voles. Four discrete vole species (Harting’s vole *Microtus hartingi,* East European vole *Microtus levis,* Thomas’s pine vole *Microtus thomasi,* and Grey dwarf hamster *Cricetulus migratorius*) comprised 40.5% (11,770 vole prey items) of the total Barn Owl prey intake. The presence and abundance of the voles varied according to underlying environmental gradients, with soil texture and type playing a major role. *M. levis* showed no significant attachments to gradients, other than a mild increase in Mollisol soils. It was syntopic in all sites with *M. hartingi,* which was the dominant and most abundant small mammal species, preferring non-arable cultivated land, natural grasslands, set-aside fields, and fallow land. *M. thomasi* was strictly present in western Thessaly and strongly associated with a sandy-clay soil texture and Alfisol soils. *C. migratorius* was the least represented vole (162 items), exclusively present in eastern Thessaly and demonstrating a stronger association with cereals, Mollisol soils, and an argillaceous-clay soil texture. This is the first study in Greece at such a large spatial scale, offering insights for pest rodents’ distribution in intensive agroecosystems and their response to environmental gradients including soil parameters.

## 1. Introduction

Rodents are globally widespread pests [1]. Voles in particular are the most common vertebrate pests in European agriculture [2], and during population outbreaks, they reach densities of up to 1000 or even 2000 individuals per hectare [3]. As a result, millions of hectares of agricultural land are damaged and millions of Euros in agricultural revenue are lost in the European Union (hereafter EU) every year [1,4,5]. In addition, rodents can also transmit numerous pathogens that cause severe symptoms, even death [6,7,8].

Although anticoagulant rodenticides were once the main method used by EU farmers to protect their farms, products that include anticoagulant rodenticidal compounds are not available anymore in most countries of the EU for plant protection purposes [8]. Compounds that include phosphine gas are approved, but due to their high acute action and toxicity there is reluctance towards their application by farmers and authorities. Consequently, that leaves space to either suffer more damage to farm production or use illegal chemical compounds, which create further secondary poisoning and threaten biodiversity [9,10]. Still, from the emerging spectrum of alternative vole management methods (ploughing, repellents, traps, fences, and flooding), none are considered suitable for large-scale agriculture [8]. Biocontrol with predatory birds, such as the Barn Owl, also serves as a large-scale solution [11]. Labuschagne [11] initially claimed that there is not enough quantitative experimental data to support that, pinpointing the need to include more statistical rigor to detect and measure change in rodent pest species abundance. However, recent research provides such evidence quantifying the positive effect of Barn Owl predation upon pest rodent abundances [12,13].

On the other hand, rodents—especially voles—also play a crucial role in the ecosystem as important prey to both generalists and specialist top predators, including both terrestrial carnivores and raptors [14]. Consequently, voles demonstrate a contradictory nature in their ecosystems and agriculture, i.e., (i) they are important and often optimum prey for top predators in nature, and (ii) they have a triple negative impact on human lives (damage to crops, annual loss of revenue, and transmission of pathogens and zoonoses). As such, in order to propose any mitigation and management measures for pest voles in agricultural ecosystems—mechanical, chemical, biocontrol, or combined—a number of initial fundamental questions must first be answered: (i) What is the distribution range of each vole species in an agroecosystem? (ii) Are there concrete spatial patterns in vole abundance and distribution range? (iii) Do different vole species respond distinctly to environmental gradients?

In extensive agricultural ecosystems, the live trapping of small mammals cannot answer large-scale questions since the method is time demanding, applied mainly in small scale monitoring studies even in multiannual efforts, and mostly focuses on population dynamic assessments [15]. On the contrary, Barn Owl diet analysis has been proven to be an ideal proxy to assess the composition, distribution, and abundance patterns of small mammals on large geographical scales [16,17]. The Barn Owl is considered the most widespread and anthropofilous avian species, present in all continents except for the polar and desert regions [18]. In Europe, the Barn Owl is a least-concern species with a population range of mature individuals 164,000 to 356,000, indicating, though, a decreasing population trend [19]. In Greece, the species’ population is estimated at 3000 to 6000 pairs [20]. Although the Long-eared owl *Asio otus* is a nocturnal raptor that also preys on voles [21], it is not found on the whole extension of Thessaly plains due to the lack of forest patches in the agricultural plain that could offer roosting sites. The *Asio otus* population in Thessaly is also not stable due to migration patterns, with a limited number of individuals staying all year long, whereas the Barn Owl is resident throughout the year and found in various villages in the agricultural plains. 

Our study was conducted in the Thessaly plains of central Greece, which is the largest and most intensively managed agroecosystem in the country, at approximately 5000 sq. km. Thessaly is also a stronghold for the Barn Owl population in the country, possibly hosting the largest part of its population [22]. Vole outbreaks cause severe yearly losses in the agricultural production of Thessaly plains. Therefore, the main aim of our study was to explore and identify the distribution patterns of voles and their individual responses to the studied environmental gradients. Through the Barn Owl diet spectrum, we can establish first-level knowledge of the attachment of voles to concrete underlying environmental parameters in the agricultural landscape and identify the areas where each pest rodent species appears with higher abundances. These insights may lead to concrete management discussions and decisions. 

## 2. Materials and Methods

### 2.1. Field Monitoring and Data Collection

From 2000 to 2002, the exhaustive monitoring of natural Barn Owl breeding sites (old buildings) was carried out in 300 villages on the Thessaly agricultural plains in central Greece. Thessaly is the largest and most intensive agricultural ecosystem in Greece and is considered as a stronghold for the Barn Owl population in the country. A total of 31 high-fidelity breeding sites were finally selected, where the minimum distance between adjacent sites was larger than 4 km. In early April 2003, older prey items, prey remains, and pellet debris were removed from all 31 selected locations. Four seasonal pellet samplings were then performed at all 31 sites from 2003 to 2005 as follows: (i) in September 2003, representing the 2003 Barn Owl breeding period; (ii) in March 2004, representing the 2003–2004 Barn Owl non-breeding period; (iii) in September 2004, representing the 2004 Barn Owl breeding period; and (iv) in March 2005, representing the 2004–2005 Barn Owl non-breeding period. The samplings comprised a total of 10,065 Barn Owl pellets, which were analyzed according to the dry method [23,24]; the recovered small mammal remains (skulls and bones) were identified based on morphology and measurements [25,26,27,28,29,30,31,32,33]. The total number of prey items that were recovered from pellet analysis and the identification process that belong to each different small mammal species comprises the “absolute frequency—n” value (Table 1). Dividing n values for each species with the total number of identified prey items produces the “relative frequency—n%” value. Finally, the “biomass contribution—gr%” of each small mammal species in Barn Owl diet was calculated using standard literature references [29,30,31,32,33] by multiplying the total number of prey items for each species with its respective average biomass and dividing with the total calculated biomass in the Barn Owl diet.

### 2.2. Datasets

The abundance and distribution patterns of small mammals, specifically voles, were the variables under study. Therefore, a response variable matrix was constructed including the prey items’ relative frequency for each identified species, per season and breeding site. The response matrix included each one of the 31 breeding sites as rows, for each of the four sampling seasons, giving a total of 124 rows. The columns comprised each different identified small mammal species, giving a discrete relative frequency value in each cell.

For the construction of the predictor dataset, we calculated the extension of land occupied by the following environmental variables within a 2 km radius around each Barn Owl breeding site: With respect to “Soil Parameters”, we calculated (i) soil types (Alfisol, Entisol, Inceptisol, Mollisol, and Vertisol) and (ii) Soil texture (sandy-clay or argillaceous-clay). With respect to “Agricultural Cultivations”, we calculated (i) cereal cultivations (wheat, barley, oat, and corn) and (ii) industrial cultivations (cotton, tobacco, and sugar beets). In terms of “Land Uses”, we estimated (i) arable cultivated land (annual pastures, cereals, and industrial cultivations); (ii) non-arable cultivated land (tree cultivations, orchards, vineyards, multiannual pastures, and vegetables); (iii) irrigated cultivated land; (iv) non-irrigated cultivated land; (v) other land uses (fallow land, set-aside fields, hills, natural grasslands, and urban settlements); and (vi) total cultivated land. Due to the absence of accurate satellite photos at the time of the study and the difficulty of applying remote sensing identification to seasonal crops and land uses in the early 2000s without an appropriate digital cartographic base, all crop and land use variables were calculated through a combination of: (i) agricultural datasets provided by the regional statistical service office from each prefecture in Thessaly; (ii) classical 1:5000 printed maps offered by the topographic services in each respective prefecture of Thessaly; (iii) in situ GPS point verification; and (iv) soil cartography in 1:20,000 printed maps provided by the Institute of Cartography and Soil Taxonomy—Hellenic Agricultural Organization Demeter (formerly the National Agricultural Research Foundation (NAGREF)).

### 2.3. Data Analysis

Due to the high correlation between environmental variables, factor analysis was applied to reduce both the size of the predictor dataset and produce new, non-correlated factors combining the original environmental variables [34,35,36,37]. Factor analysis was applied to two separate predictor datasets. One including crops and land uses, and the other including soil types and soil texture, in order to produce new factors that combined independent variables of same nature [37]. The predictor datasets imported to factor analysis (crops, land uses and soil types, and soil texture) included the land extension (%) occupied by each variable within a 2 km radius around each site for each sampling season, similar to the response matrix. The percentages were transformed using the arcsine method, and factor analysis was applied. Ιn order to decide which factor loadings actually defined each one of the new factors, a correlation matrix was constructed between the transformed independent variables and the produced factor scores, and the Bonferroni correction was applied to each one of the produced correlations separately; the remaining significant correlations were the factor loadings (original variables) that actually explained the new factors.

Once the collinearity problem was solved through factor analysis, the abundance and distribution patterns of voles in Thessaly were explored using ordination techniques, since our objective was to study the continuous change in community composition along various environmental gradients [38]. Ordination analysis was carried out using the Canoco software, version 5.0 for Windows [38,39]. A principal component analysis (PCA) was firstly applied to the response matrix as an indirect gradient analysis to indicate whether linear or unimodal methods should be used; then, a redundancy analysis (RDA) was applied to both the response and predictor datasets, with the predictor dataset now including the new respective factor scores and values. RDA, as a constrained ordination technique, creates new axes in two dimensions from the multidimensional space of predictor variables (environmental gradients), which correspond to the directions of greatest variability in the response variables within the datasets that can be best explained by the environmental variables.

We then applied Monte Carlo permutations to the samples (which are the “rows” or else “cases”) in the “environmental” dataset, while the corresponding values in the “species” matrix were kept intact. Monte Carlo permutations were used to evaluate the test statistics for each and every one of the independent variables included in the “environmental” dataset, both for their conditional and simple/marginal effects, adjusting with the false discovery rate to avoid the probability of type I error [40]. In terms of the whole predictor dataset, a conditional effect is the effect that each explanatory (environmental) variable has on the response variables, beginning from the most important. On the other hand, a simple/marginal effect is the amount of variability within the species dataset that would be explained by a constrained ordination model, when using one standalone environmental variable as the sole explanatory variable. The percentage contribution of each predictor variable was also calculated using a forward selection process.

The individual responses of each one of the four vole species were tested upon each predictor–environmental gradient with the application of generalized linear models using the Canoco software, and the best-fit model (first- or second-order polynomial model) was chosen with the Akaike criterion (AIC). A graphical visualization of the voles’ distribution and abundance in Thessaly was realized using the inverse distance weighting interpolation method [41,42] and individual response curves from the “Graph Attributes” in Canoco 5. 

## 3. Results

From a total of 10,065 Barn Owl pellets, we recovered 29,061 prey items, out of which 28,475 (97.97%) were small mammals. A total of 15 small mammal species were identified in the Thessaly plains, including four different vole species: *M. hartingi*, *M. levis*, *M. thomasi,* and *C. migratorius*. Voles dominated the Barn Owl diet, comprising 40.50% of the total prey items (Table 1).

The application of factor analysis on the predictor datasets produced three main factors in the “Agricultural Crops and Land Uses” group, explaining 92% of the variance, and three main factors in the “Soil Types and Soil Texture” group, which explained 80% of the variance, according to the Kaiser criterion in both cases. A total of 124 factor scores were produced for each new factor (4 samplings, 31 sites). Once the Bonferroni corrections were applied, the new factors were defined through their respective significant factor loadings and renamed according to their new attributes. From the “Agricultural Crops and Land Uses” group, factors 1, 2, and 3 were renamed intensive cultivations, land uses, and arable land, respectively, while from the “Soil Types and Soil Texture” group, factors 1, 2, and 3 were renamed soil texture; soil type E, M, and V; and soil type I and V (Table 2). 

We initially applied an indirect gradient analysis (PCA) to the response matrix, which demonstrated that linear methods should be used in continuation to produce a constrained model, since the largest gradient’s value was less than 3 (2.2 SD units long) (Table 3). Therefore, a direct-gradient redundancy analysis (RDA), or constrained analysis, was applied to both the response and predictor matrices. All produced canonical (constrained) axes were measured as the percentage of explained variance and permutation resulted in a significant constrained model (first axis: pseudo-F = 1.4, *p* = 0.0099; all axes: pseudo-F = 4.4, *p* = 0.0099). This suggested that the constrained environmental axis could explain the variability within the response matrix, where the two first constrained axes explained almost 70% of the variability in the response dataset (Table 3).

The conditional effects of all six underlying environmental gradients, as derived from the application of Monte Carlo permutations, demonstrated the significant participation of all gradients in explaining part of the total variation, where the Inceptisol and Vertisol soil gradients “Soil Type I and V” were the least powerful. Similarly, the simple/marginal effects indicated that the environmental gradient “Soil Type I and V” was the only predictor variable without a significant effect when used as the sole explanatory variable to explain total variation (Table 4).

**Figure 1:**
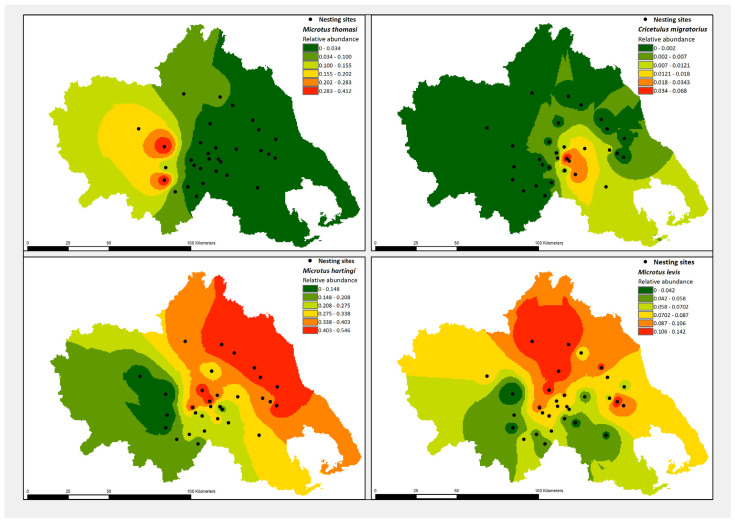
Distribution patterns of four vole species (*M. hartingi*, *M. levis*, *M. thomasi*, and *C. migratorius*) in the agroecosystems of Thessaly, central Greece, based on the spatial interpolation of values from all 31 Barn Owl nesting sites, from which pellet samplings and pellet analysis were carried out. Interpolation was based on the inverse distance weight model (IDW), which determines cell values using a linearly weighted combination of a set of measured values of sample points, where the weight is a function of the inverse distance from the output cell location. Upper left panel, *M. thomasi* distribution patterns; upper right panel, *C. migratorius* distribution patterns; lower right panel, *M. hartingi* distribution patterns; lower right panel, *M. levis* distribution patterns.

Each of the four vole species in our study, *M. hartingi*, *M. levis*, *M. thomasi,* and *C. migratorius*, demonstrated different distribution patterns in the Thessaly plains. According to the interpolation performed using the inverse distance weighted method (IDW) and the geostatistical analyst extension to ArcMap (Figure 1), the voles presented different strongholds within the agricultural ecosystem.

**Figure 2:**
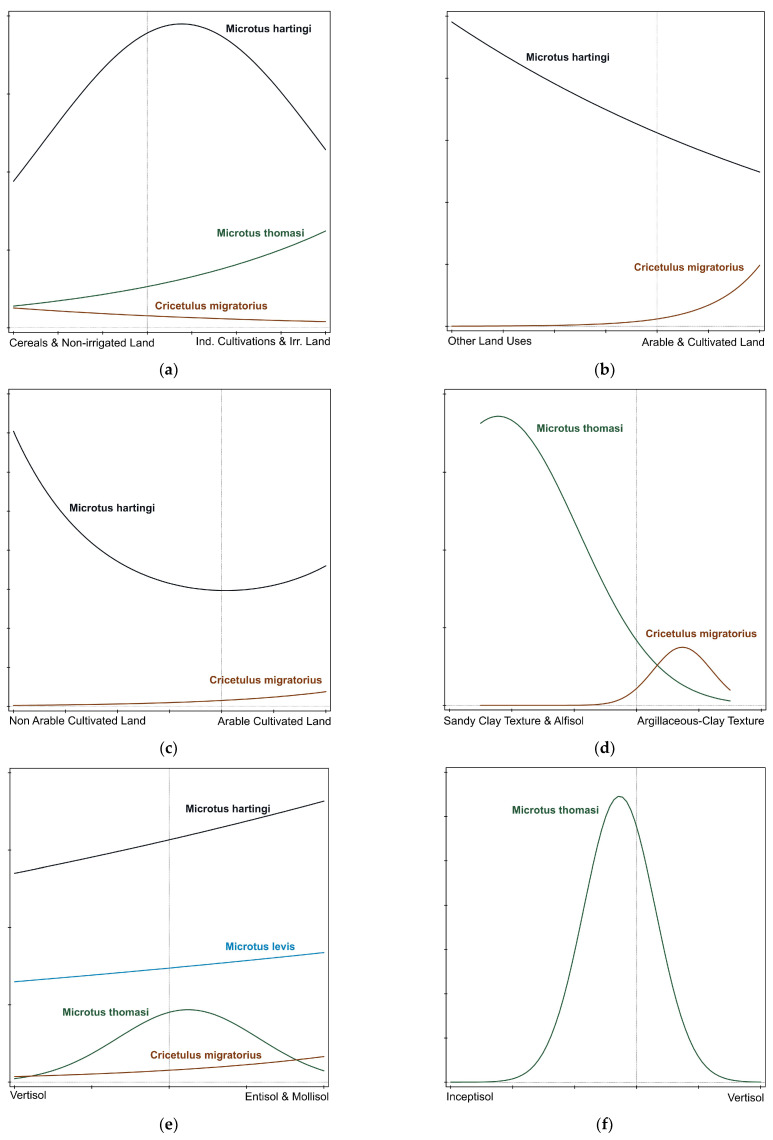
Individual response patterns for each vole species to the six discrete environmental gradients. Only responses that fitted a first- or second-order polynomial model are included in the graphs. (**a**) Vole response curves for the “Industrial Cultivations Gradient”; (**b**) vole response curves for the “Land Uses Gradient”; (**c**) vole response curves for the “Arable Land Gradient”; (**d**) vole response curves for the “Soil Texture Gradient”; (**e**) vole response curves for the “Soil Types EM and V Gradient”; (**f**) vole response curves for the “Soil Types I and V Gradient”.

Different responses were recorded for each vole with respect to the underlying environmental gradients. The best-fit response model was appointed as a first-order or a second-order polynomial model based on the Akaike criterion and the application of GLM analysis for each case (Table 5). The visualization of the response model for each vole species was realized with the Canoco 5 software and species response curves application (Figure 2).

## 4. Discussion

Small mammals are considered as an ideal taxonomic group to be used as the model species for addressing questions at different spatial scales, ranging from small plots to landscapes [43,44]. In the current study, we used the diet of Barn Owls as a proxy on a large spatial scale, covering more than 3,000 sq. km. in the central Greece plains in order to explore the response of four vole species to discrete environmental gradients. Similar studies have proved Barn Owl diet to be an optimum proxy, especially in open low-altitude areas, but also used in mountainous ecosystems [16,17]. The Barn Owl diet in the agroecosystem of Thessaly mainly consisted of four species of voles, which formed 40.5% in relative frequency terms and 50.4% of the total consumed biomass. 

The most abundant species of vole was *M. hartingi*, which was present in 30 out of 31 sampling sites and demonstrated a relative frequency of occurrence among sites ranging from 4.22% to 48.55%, with higher abundances in the northeastern Thessaly plains, according to the IDW interpolation method. *M. hartingi* is an endemic species of the Balkan Peninsula that prefers natural grasslands, well-drained meadows, and sparse vegetation, exhibiting minimum tolerance to ploughing and fields with arable cultivations, which destroy its shallow nests near to the surface [32,45,46]. Similarly, in Thessaly, the species appears to strongly avoid both cereal and intensive cultivated crops, where its abundance increases when these crops are minimized within the complex mosaic of cultivations that comprise the sampled sites and other type of crops begin to emerge. Moreover, the species also presents a clear distribution pattern in avoiding heavily arable fields, where it is significantly more abundant in different types of land such as set-aside fields, fallow land, multiannual pastures, and natural grasslands, which comprise the negative values of the factor “Land Uses”. Although all the *Μ. hartingi* responses fitted significant models, the strongest fit appeared for the second-order polynomial model with respect to the “Intensive Cultivations” gradient, according to which the species avoids both ends of the gradient and shows a peak in the middle of the gradient where intensive cultivations and cereal crops are minimized. 

In the same spatial context, *M. levis* was also present in the same 30 sampling sites and was found to be syntopic with *M. hartingi* on all occasions, but with much lower abundances. The species’ relative frequency of occurrence exceeded 10% in only a few cases. Similar to the abundance patterns of *M. hartingi*, *M. levis* presents higher numbers in eastern and northern locations, with a higher concentration in the northern Thessaly plains. *M. levis* is a Palearctic species with a distribution range that also includes the Balkan Peninsula [47]. The species’ habitat requirements in its Palearctic distributional range are meadows, agricultural land, and windbreaks [47,48], and in its eastern range in Anatolia it also prefers tall and herbaceous vegetation and especially wet and marshy places [32]. In Thessaly, it was syntopic with *M. hartingi*; however, due to its uniform and low occurrences in all sites, it did not fit any significant response model along the measured environmental gradients. Only a weak, non-significant first-order polynomial response model appeared for the gradient “Soil EM and V”; the species showed a mild increase towards Entisol and Mollisol soil types. 

Both Entisol and Mollisol soil types have positive values in the “Soil EM and V” gradient. Entisol soil types are sandy mineral soils that lack developed soil horizons; they may have thin surface horizons with some accumulation of organic matter, but they lack enough alteration of parent materials to form other horizons [49]. Entisols are generally found in young landscapes where time has not been sufficient for soils to develop and are low in organic matter, natural fertility, and water-holding capacity [49]. On the other hand, Mollisols are mineral soils with thick, dark surface horizons that are relatively high in organic matter and have high base saturation [50]. Mollisols are mainly found in grassland ecosystems. They are characterized by a thick, fertile, dark surface horizon, known as a mollic epipedon, which results from the long-term addition of organic materials derived from plant roots, and typically have a soft, granular soil structure between 60 and 80 cm deep [50]. It is possibly due to the Mollisol soil types, the “grassland soils”, that an increase was also noted in both *M. hartingi* and *M. levis*, which prefer grassland sites, although this increase was not significant. Vertisols are heavy clay soils that form deep wide cracks from the surface and downwards when they dry out, a phenomenon that occurs regularly [51]. They are dark-colored soils that are typically found on level or mildly sloping topography. Vertisols occupy the negative values in the “Soil EM and V” gradient. All four vole species showed decreases in terms of their presence and abundance towards the areas of the Thessaly plains with Vertisol soils. 

The other two vole species in our study, *M. thomasi* and *C. migratorius*, occupy different areas in Thessaly in comparison to the abundant and dominant *M. hartingi* and *M. levis*, and present an allopatric pattern between them. *M. thomasi* demonstrated higher abundances strictly in western Thessaly, with its presence indicated in no more than 12 sites, whereas the exact opposite pattern was observed for *C. migratorius,* which was present in 10 sites strictly in eastern Thessaly, being syntopic on only one occasion with *M. thomasi*. 

*M. thomasi* is the most fossorial species of all Balkan Microtus voles [52], and its distribution is restricted to deeper soil, which can easily be excavated and sustains tunnels that can be maintained over time [30,52]. It is also an endemic species to the southwestern Balkans, occupying mainly southern locations in Greece, starting from central areas and including the whole of Peloponnesus [52]. The species’ response models were mainly attached to soil types and soil texture. Specifically, *M. thomasi* strongly avoided all areas that were dominated by Vertisol, Inceptisol, Entisol, and Mollisol soil types, showing a clear preference for sites dominated by the Alfisol soil type and a sandy-clay texture. 

In contradiction to *M. hartingi*, *M. thomasi* presented higher numbers in areas with industrial cultivations and intensive irrigation schemes, demonstrating that it may not be affected by cultivations which require ploughing arable practices (possibly since it is a highly fossorial species) but chooses its habitat mainly according to soil properties. It is also possible that the Alfisol soil type, which includes mineral soils relatively low in organic matter and relatively high in base saturation, combined with a sandy-clay texture is ideal for the high fossorial habits of *M. thomasi* in Thessaly. It is the only vole that had a highly significant response model, indicating an increase towards Alfisol soil types and soil with a sandy-clay texture, although sandy soils have been recorded in the literature to be avoided by other rodent species [2]. Land uses and arable land as environmental gradients did not affect its habitat selection nor its distribution range.

The least represented vole species in our study was *C. migratorius*, present in only 10 out of 31 sampled sites with low percentages never exceeding 7% of the relative frequency of occurrence. It is a species with a Palearctic distribution range that is confined to the Balkan Peninsula with few isolated and small populations [53]. The species’ subspecific status in the Balkans is uncertain since it is the most diverse among the hamsters’ group. In Thessaly, it was considered to be present only in a southern part of the city of Farsala, first recorded by Niethammer and Krapp [29,30] and later reviewed by Vohralik [53]. The results of our study demonstrate that the species has a broader distribution in the Thessaly plains, confined strictly to eastern locations. 

The *C. migratorius* response model demonstrated a very strong correlation to the “Soil Texture” gradient indicating a clear increase towards agricultural areas with a mainly argillaceous-clay texture. In parallel, it was the most unique of all recorded vole species, demonstrating significantly higher numbers in areas dominated by Entisol and Mollisol soil types. *M. hartingi* and *M. levis* also showed increases towards Mollisol soils, or the “grassland soils”; however, the response was not significant. Its distribution range and habitat selection were independent of both irrigation schemes and industrial crops, yet it presented a preference towards sites with arable cultivated land and cereals, a distribution pattern opposite to that of *M. hartingi* and *M. thomasi*. The original habitats of *C. migratorius* include dry grasslands, steppes, and semideserts [54]; however, it is also present in agricultural areas and gardens and is also often synanthropic [30,53,54]. It exhibits a level of habitat plasticity as a species [54], which could possibly explain its presence in types of land and crops avoided by other vole species in Thessaly. 

To our knowledge, this is the first study using Barn Owl diet analysis with representative samples from such a spatial extension in an agricultural ecosystem of Greece; moreover, it is the first study in the country to analyze more than 10,000 prey items seasonally recovered throughout a period of two consequent years. In our study, soil texture and soil type appeared to be the most important variables. *M. thomasi* and *C. migratorius* were the two species whose distribution was mainly defined by soil texture with allopatric patterns. Similarly, in studies conducted in Europe [2] as well as in Neotropical regions, India, and Tanzania, it has been shown that soil texture clearly defines small mammals’ choice of habitat and distribution [55,56,57,58]. We suggest incorporating soil texture and soil types in future studies as possible major drivers of habitat suitability, distribution patterns, and abundance responses of voles in agricultural plains as part of the predictor datasets. The results can offer important information in combination with landscape characteristics and land uses in the design of tailor-made management and mitigation measures against species-specific population outbreaks, taking into consideration the specifics of fossorial ecology with respect to soil types and texture for each different species.

## 5. Conclusions

Small mammals represent a crucial element of terrestrial ecosystems. They serve as prey, pests, and are also mechanics of the soil that physically shape their habitats [59]. Their distribution and abundance patterns are affected by several major groups of environmental characteristics and parameters [60]. Nonetheless, static site characteristics, such as soil types and soil texture, are often not included in the literature to explain the distribution of small mammals and their abundance response patterns. Soil texture and soil types appeared to be the most important variables in our study. Mollisols appear to have a non-significant but still apparent effect on increasing the presence of *M. hartingi* and *M. levis*, while Vertisols are avoided. The most abundant pest vole in Thessaly, *M. hartingi*, appears to have high concentrations in non-arable cultivations and other land types (set-aside land, fallow land, multiannual pastures, and natural grasslands), from which it usually emerges during outbreaks and causes damages to adjacent crops. *M. levis* appears to be unattached to any of the studied environmental gradients and syntopic/sympatric with *M. hartingi*. The possibility of *M. levis* being sub-dominant to the dominant *M. hartingi*, exploiting the same niche with lower numbers and thus indicating no significant attachment to environmental gradients, must be corroborated further field studies.

Deep insights can be offered in terms of small mammal distribution and response patterns through Barn Owl diet analysis. Still, limitations of our study can be found in the time-consuming process of collecting and analyzing pellets and identifying small mammal bone remains recovered from pellets. Another limitation can be found in the continuous decrease in natural breeding Barn Owl sites that is noted in various countries in Europe, which can be overcome with the installment of Barn Owl nest boxes. 

## Figures and Tables

**Table 1:** Results of Barn Owl diet analysis in Thessaly plains, central Greece: absolute frequency (n), relative frequency (n%), and biomass contribution (gr%). Unidentified items are denoted as “un”.

	Absolute Frequency	Relative Frequency	Biomass
	n	n%	gr%
Bicoloured white-toothed shrew (*Crocidura leucodon*)	708	2.44%	0.72%
Lesser white-toothed shrew (*Crocidura suaveolens*)	6229	21.43%	4.80%
*Crocidura* un.	37	0.13%	0.03%
Etruscan shrew (*Suncus etruscus*)	478	1.64%	0.09%
*Soricidae*	7452	25.64%	5.64%
INSECTIVORA	7452	25.64%	5.64%
Harting’s vole (*Microtus hartingi*)	8313	28.60%	40.05%
East European vole (*Microtus levis*)	2060	7.09%	7.05%
Thomas’s pine vole (*Microtus thomasi*)	1233	4.24%	2.73%
*Microtus* un.	2	0.01%	0.01%
Grey dwarf hamster (*Cricetulus migratorius*)	162	0.56%	0.55%
*Cricetidae*	11,770	40.50%	50.39%
Yellow necked mouse (*Apodemus flavicollis*)	973	3.35%	2.63%
Western broad-toothed field mouse (*Apodemus epimelas*)	201	0.69%	0.81%
Wood mouse (*Apodemus sylvaticus*)	2024	6.96%	3.90%
*Apodemus* un.	26	0.09%	0.08%
Brown rat (*Rattus norvegicus*)	500	1.72%	19.15%
Black rat (*Rattus rattus*)	223	0.77%	4.46%
*Rattus* un.	129	0.44%	3.76%
House mouse (*Mus musculus*)	3644	12.54%	5.97%
Macedonian mouse (*Mus macedonicus*)	1375	4.73%	1.99%
*Mus* un.	99	0.34%	0.15%
*Muridae*	9194	31.63%	42.90%
Hazel dormouse (*Muscardinus avellanarius*)	50	0.17%	0.11%
*Myoxidae*	50	0.17%	0.11%
RODENTIA	21,014	72.30%	93.40%
Common pipistrelle (*Pipistrellus pipistrellus*)	2	0.01%	0.01%
*Vespertilionidae*	2	0.01%	0.01%
European free-tailed bat (*Tadarida teniotis*)	2	0.01%	0.01%
Molossidae	2	0.01%	*0.01%*
Greater horseshoe bat (*Rhinolophus ferrumequinum*)	5	0.02%	0.01%
*Rhinolophidae*	5	0.02%	0.01%
CHIROPTERA	9	0.03%	0.03%
MAMMALIA	28,475	97.97%	99.06%
House sparrow (*Passer domesticus*)	100	0.34%	0.24%
Eurasian tree sparrow (*Passer montanus*)	25	0.09%	0.05%
*Passeridae*	125	0.43%	0.29%
European greenfinch (*Carduelis chloris*)	31	0.11%	0.08%
European serin (*Serinus serinus*)	39	0.13%	0.05%
Common chaffinch (*Fringilla coelebs*)	44	0.15%	0.09%
*Fringillidae*	114	0.39%	0.22%
Corn bunting (*Milaria calandra*)	16	0.06%	0.07%
*Emberizidae*	16	0.06%	0.07%
Common blackbird (*Turdus merula*)	14	0.05%	0.13%
European robin (*Erithacus rubecula*)	26	0.09%	0.05%
*Turdidae*	40	0.14%	0.18%
Great tit (*Parus major*)	10	0.03%	0.02%
Eurasian blue tit (*Parus caeruleus*)	15	0.05%	0.02%
*Paridae*	25	0.09%	0.03%
Common starling (*Sturnus vulgaris*)	6	0.02%	0.05%
*Sturnidae*	6	0.02%	0.05%
Eurasian magpie (*Pica pica*)	2	0.01%	0.04%
Corvidae	2	0.01%	0.04%
*Corvidae*	2	0.01%	0.04%
PASSERIFORMES	328	1.13%	0.89%
Eurasian collared dove (*Streptopelia decaocto*)	3	0.01%	0.05%
*Columbidae*	3	0.01%	0.05%
COLUMBIFORMES	3	0.01%	0.05%
AVES	331	1.14%	0.94%
Meadow grasshopper (*Chorthippus parallelus*)	115	0.40%	
Migratory locust (*Locusta migratoria*)	60	0.21%	
*Acrididae*	175	0.60%	
European mole cricket (*Gryllotalpa gryllotalpa*)	9	0.03%	
*Gryllotalpidae*	9	0.03%	
Great green bush-cricket (*Tettigonia viridissima*)	9	0.03%	
*Tettigonidae*	9	0.03%	
ORTHOPTERA	193	0.66%	
Black ground beetle (*Pterostichus nigrita*)	21	0.07%	
Bronze carabid (*Carabus nemoralis*)	11	0.04%	
*Carabidae*	32	0.11%	
Horned dung beetle (*Copris lunaris*)	20	0.07%	
Common cockschafer (*Melolontha melolontha*)	10	0.03%	
*Scarabaeidae*	30	0.10%	
COLEOPTERA	62	0.21%	
INSECTA	255	0.88%	
Total Prey Items	29,061		

**Table 2:** Correlations between independent variables and factor scores produced via factor analysis for each group of predictor variables. Significant p_s_(+) are presented after the level of significance was corrected using the Bonferroni correction (a = 0.05/_(number of variables) × (number of factors)_). Significant p_s_ suggest which factor loadings (original variables) “define” the new factors and are noted as according to their significance level: *p* < 0.01—*; *p* < 0.001—**; *p* < 0.0001—***; *p* < 0.00001—****; *p* < 0.000001—*****.

1st Group	Factor 1 Intensive Cultivations	Factor 2 Land Uses	Factor 3 Arable Land	2nd Group	Factor 1 Soil Texture	Factor 2 Soil Type E, M, and V	Factor 3 Soil Type I and V
Cereals	−0.7336 **			Alfisol soil type	−0.7208 **		
Industrial cultivations	0.9380 *****			Entisol soil type		0.8441 **	
Arable cultivated land		0.9054 **	0.4128 ***	Inceptisol soil type			−0.9503 **
Non-arable cultivated land			−0.9776 **	Mollisol soil type		0.6666 *****	
Irrigated cultivated land	0.9282 *****			Vertisol soil type		−0.6750 *****	0.4700 *****
Non-irrigated cultivated land	−0.9119 *****			Sandy-clay texture	−0.9345 *****		
Other land uses		−0.9989 **		Argillaceous-clay texture	0.9463 *****		
Total cultivated land		0.9989 **					

**Table 3:** Indirect gradient analysis (PCA) only taking into account the variability of the “species” matrix (dependent variables) and direct gradient analysis (RDA) taking into account the variability of both the “species” and “environmental” (independent variables) matrices.

Principal Component Analysis (PCA)				
Axes	1	2	3	4
Eigenvalues	0.410	0.196	0.104	0.068
Cumulative percentage variance of species data	41.0	60.6	71.0	77.8
Redundancy Analysis (RDA)				
Axes	1	2	3	4
Eigenvalues	0.0689	0.0608	0.0262	0.0236
Explained variation (cumulative)	6.89	12.97	15.59	17.95
Pseudo-canonical correlation	0.6517	0.5892	0.5798	0.4996
Explained fitted variation (cumulative)	37.12	69.90	84.01	96.74

**Table 4:** Results of Monte Carlo permutations on the predictor variables. Marginal/simple effects summarize the effects of each predictor variable when used as the only explanatory variable in the model. Conditional effects are the effects of each predictor variable on the whole environmental dataset. Variables are ranked according to their significance in explaining total variation, and *p* values are corrected for the false discovery rate. Forward selection (FS) demonstrates the percentage contribution of each environmental gradient in explaining the constrained model (RDA) fitted variation during a forward selection process of including each variable in the model.

Simple/Marginal Effects					Conditional Effects					FS
Variable	Explains %	Pseudo-F	p	p adj	Variable	Explains %	Pseudo-F	p	p adj	Contribution
Soil texture	5.0	6.4	0.0099	0.01485	Soil texture	5.0	6.4	0.0099	0.0099	27%
Intensive cultivations	4.5	5.8	0.0099	0.01485	Intensive cultivations	4.3	5.8	0.0099	0.0099	23.4%
Soil E, M, V	3.0	3.8	0.0099	0.01485	Soil E, M, V	3.1	4.2	0.0099	0.0099	16.5%
Arable land	2.4	3.0	0.0099	0.01485	Land uses	2.3	3.2	0.0099	0.0099	12.3%
Land uses	2.2	2.8	0.0099	0.02376	Arable land	2.1	3.0	0.0099	0.0099	11.6%
Soil I and V	1.4	1.8	0.06931	0.06931	Soil I and V	1.7	2.5	0.0099	0.0099	9.2%

**Table 5:** Response of small mammal species to each environmental gradient. “Best fit” model selection was carried out according to the Akaike criterion (AIC) through generalized linear model analysis. Response variables that did not fit any model and were rejected through the “null model” hypothesis are not included in the table. Significant p_s_ are noted as: *p* < 0.05—*; *p* < 0.01—**; *p* < 0.001—***; *p* < 0.0001—****; *p* < 0.00001—*****. R2(%) provides a measure of explained variation, paralleling the coefficient of determination in classical regression, calculated here as the ratio of the deviance explained by the fitted model and the deviance of the null model (with no predictors) multiplied by 100. F test statistic and the following *p* estimate of the type I error rate correspond to an overall parametric test of the selected model against the null model, pooling the effect of both predictors when two predictors were present.

			Model Selection		Generalized Linear Model Results	
	R2 (%)	AIC	b_0_ + b_1_X	b_0_ + b_1_X + b_2_X^2^	F	*p*
INTENSIVE CULTIVATIONS						
Harting’s vole (*Microtus hartingi*)	15	1408.23		√	12.2	****
Thomas’s pine vole (*Microtus thomasi*)	6.4	2122.53	√		6.4	*
Grey dwarf hamster (*Cricetulus migratorius*)	3.0	794.90	√		2.6	0.10773
LAND USES						
Harting’s vole (*Microtus hartingi*)	6.4	1475.21	√		9.5	**
Grey dwarf hamster (*Cricetulus migratorius*)	12.0	728.27	√		12.5	***
ARABLE LAND						
Harting’s vole (*Microtus hartingi*)	4.8	1502.53		√	3.4	*
Grey dwarf hamster (*Cricetulus migratorius*)	4.3	655.43	√		4.0	*
SOIL TEXTURE						
Thomas’s pine vole (*Microtus thomasi*)	34.8	1575.52		√	20.7	*****
Grey dwarf hamster (*Cricetulus migratorius*)	29.2	610.51		√	22.6	*****
SOIL TYPES E, M, AND V						
Harting’s vole (*Microtus hartingi*)	2.4	1510.84	√		3.5	0.06401
East European vole (*Microtus levis*)	2.2	878.81	√		3.3	0.07313
Thomas’s pine vole (*Microtus thomasi*)	9.4	2096.70		√	4.9	**
Grey dwarf hamster (*Cricetulus migratorius*)	4.4	781.97	√		4.3	*
SOIL TYPES I AND V						
Thomas’s pine vole (*Microtus thomasi*)	23.3	1798.84		√	14.9	*****

## Data Availability

Not applicable.
